# Overexpression of rck/p54, a DEAD box protein, in human colorectal tumours

**DOI:** 10.1038/sj.bjc.6690441

**Published:** 1999-05

**Authors:** Y Nakagawa, H Morikawa, I Hirata, M Shiozaki, A Matsumoto, K Maemura, T Nishikawa, M Niki, N Tanigawa, M Ikegami, K Katsu, Y Akao

**Affiliations:** The Second Department of Internal Medicine and Department of General and Gastroenterological Surgery, Osaka Medical College, Osaka, Takatsuki, 569-8686, Japan; Clinical Service of Pathology, Jikei University Hospital, Tokyo, Minato-ku, 105-0031, Japan; Gifu International Institute of Biotechnology, Mitake-cho, Kani-gun, Gifu, 505-0116, Japan

**Keywords:** rck/p54, colorectal tumour, DEAD box protein/RNA helicase, overexpression

## Abstract

The *RCK* gene is a target of the t(11;14)(q23;q32) chromosomal translocation observed in human B-cell lymphoma, and the overexpression of its protein (rck/p54) by the translocation was shown to cause malignant transformation. The rck/p54 protein belongs to the DEAD box protein/RNA helicase family, which has a variety of functions such as translation initiation, pre-mRNA splicing and ribosome assembly. The expression of rck p54 in colorectal adenocarcinoma cells was examined by immunohistochemistry and Western blot analysis. The rck/p54 protein was found to be overexpressed in tumour tissues resected from 13 (50%) out of 26 cases of colorectal adenocarcinomas and two out of two (100%) cases of colonic severe dysplastic adenomas. In view of activities of rck/p54 determined in other tissue types, we suggest that rck/p54 may contribute to the cell proliferation and carcinogenesis at the translational level in the development of colorectal tumours. © 1999 Cancer Research Campaign


*British Journal of Cancer* (1999) **80**(5/6), 914–917
© 1999 Cancer Research Campaign
Article no. bjoc.1998.0441

### Overexpression of rck/p54, a DEAD box protein, in human colorectal tumours

**Y Nakagawa[1], H Morikawa[1], I Hirata[1], M Shiozaki[1], A Matsumoto[1], K Maemura[1], T Nishikawa[1], M Niki[2], N Tanigawa[2], M Ikegami[3], K Katsu[1] and Y Akao[4]**

[1]The Second Department of Internal Medicine and [2]Department of General and Gastroenterological Surgery, Osaka Medical College, Daigaku-cho, Takatsuki, Osaka 569-8686, Japan; [3]Clinical Service of Pathology, Jikei University Hospital, 3-19-18 Nishi-Shinbashi, Minato-ku, Tokyo 105-0031, Japan; [4]Gifu International Institute of Biotechnology, Mitake-cho, Kani-gun, Gifu 505-0116, Japan

**Summary** The *RCK* gene is a target of the t(11;14)(q23;q32) chromosomal translocation observed in human B-cell lymphoma, and the overexpression of its protein (rck/p54) by the translocation was shown to cause malignant transformation. The rck/p54 protein belongs to the DEAD box protein/RNA helicase family, which has a variety of functions such as translation initiation, pre-mRNA splicing and ribosome assembly. The expression of rck p54 in colorectal adenocarcinoma cells was examined by immunohistochemistry and Western blot analysis. The rck/p54 protein was found to be overexpressed in tumour tissues resected from 13 (50%) out of 26 cases of colorectal adenocarcinomas and two out of two (100%) cases of colonic severe dysplastic adenomas. In view of activities of rck/p54 determined in other tissue types, we suggest that rck/p54 may contribute to the cell proliferation and carcinogenesis at the translational level in the development of colorectal tumours.

**Keywords:** rck/p54; colorectal tumour; DEAD box protein/RNA helicase; overexpression

The *RCK* gene was cloned through the study of the t(11;14)(q23;q32) translocation observed in human B-cell lymphoma cell line RC-K8 (Akao et al, 1991). This gene was found to have been broken at its first intron and fused to an immunoglobulin heavy chain gene (IgH) by a t(11;14) translocation. Its protein (rck/p54) belongs to the DEAD box (D-E-A-D is the single letter code of Asp-Glu-Ala-Asp) protein/RNA helicase family, which has a variety of functions such as translation initiation, pre-mRNA splicing and ribosome assembly (Akao et al, 1992). The rck/p54 protein of 472 amino acids is a 54-kDa cytoplasmic protein and is overexpressed due to the fusion to the IgH gene in the same manner as c-*myc* in t(8;14) or *bcl*-2 in t(14;18) (Rabbitts, 1994; Akao et al, 1995). Moreover, we previously found that the expression of rck/p54 is very poor in brain, skeletal muscle and lung tissues, but is significant in tumours that originated from these tissues (Akao et al, 1995). These findings suggest that rck/p54 may be linked to cell proliferation and malignant transformation.

Recently, the *Xenopus* rck/p54 homologue Xp54, which is homologous to rck/p54 by 94%, was shown to possess RNA helicase activity. It was speculated that Xp54 may be necessary for the efficient translational recruitment of stored mRNA or enhance translation of mRNA at a time when large quantities of product are required in *Xenopus* oogenesis (Ladomery et al, 1997). It was considered that some of the DEAD box protein/RNA helicase family work on mRNAs, which have stem and loop tertiary structure, and make them change to straight resulting in facilitation of translation. The best characterized DEAD box protein, eIF-4A plays a central role in translation initiation together with two other factors, p220 and the cap-binding protein eIF-4E. In cells, eIF-4A exists in a free form or in the eIF-4F complex, which complex interacts with mRNA to facilitate its translation (Rosen et al, 1990). It was also reported that Ded1p, a yeast DEAD box protein, is required for the initial step of translation (Chuang et al, 1997). Thus, rck/p54 supposedly facilitates the translation of mRNA(s) of genes for cell proliferation and malignant transformation.

Based on these findings, we examined the expression of rck/p54 protein in the colorectal tumours by immunohistochemistry and Western blot analysis. It is of interest that we found rck/p54 to be overexpressed in approximately 54% of the colorectal tumours studied.

*Received 16 June 1998*
*Revised 24 August 1998*
*Accepted 11 September*

*Correspondence to*: Y Akao

### MATERIALS AND METHODS

### Tissue preparation

All human tissue samples (primary colorectal adenocarcinoma, adjacent normal colorectal mucosa from specimens resected for carcinoma and adenoma) were specimens obtained with informed consent from patients who were undergoing surgical operations or biopsies at Osaka Medical College, Takatsuki, Osaka, between 1996 and 1998. We examined 26 cases of colorectal adenocarcinomas and two cases of colonic adenomas (severe dysplasia). All tissues were divided into cancerous and normal parts. For each tissue sample, a small part was applied for Western blot analysis, and the remainder was fixed in 10% buffered formaldehyde solution and embedded in paraffin. For immunohistochemical study, five series of 4-μm-thick sections were prepared. One section was stained with haematoxylin and eosin and reviewed by two pathologists to confirm the diagnosis including pathological prognostic factors such as tumour type, size and stage. The other sections were used for analysing the expression of rck/p54 and mutant p53 proteins. Table 1 shows the clinical characteristics of the 28 cases

**Table 1** Immunohistochemical expression of rck/p54 and p53 and clinicopathologic features

| | | | | | | | | Immunohistochemical staining | |
|---|---|---|---|---|---|---|---|---|---|
| Case | Age | Sex[a] | Size[b] | Depth[c] | Site[d] | Stage[e] | Growth[f] | p53[g] | rck/p54[h] |
| O-1 | 73 | M | 12 | SM | R | A | PG | D | – |
| O-2 | 50 | M | 5 | SM | S | A | NPG | D | – |
| O-3 | 44 | M | 5 | SM | R | A | NPG | – | – |
| O-4 | 56 | M | 12 | SM | R | A | PG | D | 2+ |
| O-5 | 38 | M | 11 | SM | T | A | PG | – | – |
| O-6 | 74 | M | 7 | SM | T | A | NPG | – | 1+ |
| O-8 | 43 | M | 23 | SM | R | C | NPG | D | 1+ |
| O-11 | 73 | M | 8 | SM | S | A | PG | D | – |
| O-14 | 65 | F | 10 | SM | S | A | PG | D | 2+ |
| O-16 | 64 | M | 18 | SM | S | A | PG | – | 1+ |
| O-20 | 81 | M | 22 | SM | T | A | PG | – | – |
| O-24 | 67 | M | 20 | SM | R | A | PG | – | – |
| O-26 | 82 | M | 7 | SM | R | A | PG | D | 1+ |
| O-31 | 64 | M | 20 | SM | A | A | PG | – | – |
| O-33 | 60 | M | 25 | SM | R | A | PG | D | 2+ |
| O-34 | 61 | F | 27 | SM | R | A | PG | – | 1+ |
| O-35 | 69 | M | 4 | SM | R | A | NPG | FD | – |
| O-40 | 66 | M | 8 | SM | S | C | NPG | FD | 2+ |
| O-42 | 70 | F | 18 | SM | D | C | PG | – | 1+ |
| O-43 | 54 | M | 13 | SM | A | C | NPG | – | – |
| O-44 | 80 | M | 26 | SM | D | C | NPG | – | – |
| O-45 | 57 | M | 17 | SM | S | C | NPG | D | 2+ |
| O-46 | 64 | M | 10 | SM | S | A | NPG | FD | – |
| O-71 | 64 | F | 59 | MP | T | A | 2 | D | 1+ |
| O-72 | 53 | F | 27 | MP | R | A | 2 | D | – |
| O-79 | 65 | M | 30 | MP | R | A | 2 | – | 1+ |
| O-80 | 53 | F | 16 | M | A | A | PG | – | 2+ |
| O-81 | 53 | F | 22 | M | C | A | PG | – | 1+ |

[a]M, Male; F, female. [b]Diameter in mm. [c]M, adenoma (severe dysplasia); SM, submucosa; MP, muscularis propria. [d]Location of tumour; C, caecum; A, ascending colon; T, transverse colon; D, descending colon; S, sigmoid colon; R, rectum. [e]Dukes' system. [f]Growth type: PG, polypoid growth; NPG, non-polypoid growth; 2, 2type advanced cancer. [g]D, diffuse; FD, focal diffuse; –, no overexpression. [h]Overexpression; 2+, positive (tumour » normal); 1+, positive (tumour > normal mucosa); –, no overexpression.

tested, including Dukes′ staging system, differentiation grade and types of growth.

### Immunohistochemical staining

The 4-μm sections from paraffin-enbedded tissues were mounted on poly-L-lysine-coated slides. They were then deparaffinized in xylene and dehydrated with graded ethanol. Endogenous peroxidase activity was blocked with 0.2% (w/v) sodium azide containing in 0.3% (v/v) hydrogen peroxide in graded methanol for 60 min. In the case of rck/p54 protein, after having been washed in 0.01 M phosphate-buffered saline (PBS), the section was blocked for non-specific binding with Avidin/Biotin Blocking solution (Vector Laboratories, Inc., CA, USA). The sections were then incubated with rabbit polyclonal anti-rck/p54 antibody diluted at 1:200 in a moist chamber overnight at 4°C. In the case of p53 protein, the sections were incubated with anti-p53 monoclonal antibody (BP53-12, Japan Tarner, Ltd., Osaka, Japan) diluted at 1:25. After having been washing in 0.01 M PBS, the sections were subsequently incubated for 30 min at room temperature with biotinylated goat anti-rabbit immunoglobulin and biotinylated goat anti-mouse immunoglobulin for rck/p54 and p53 protein expression respectively. After another wash in PBS, peroxidase-conjugated avidin (Vector Stain Elite ABC Kit, Vector Laboratories, Inc., CA, USA) was applied, and the sections were again incubated for 30 min. In both cases, after washing out of the excess complex, the localization of immunoreactive complexes was visualized by incubation of the sections for 5–10 min in 0.05 M Tris–HCl (pH 7.6) containing 0.02% (w/v) 3-3′-diaminobenzidine tetrahydrochloride and 0.03% (v/v) hydrogen peroxide. A negative control section, to which normal rabbit preimmune serum was applied in slides for rck/p54 expression, was included in each staining. Counter staining was performed with haematoxylin.

### Western blot analysis

Fresh tissues were homogenized in lysis buffer (2 × PBS, 0.1% sodium dodecyl sulphate (SDS), 1% Nonidet P-40, 0.5% sodium deoxychlorate, and 0.1 mM phenylmethanesulphonyl fluoride). The homogenized samples were centrifuged, and the clear supernatant was used as protein lysate. The protein concentration was determined by the method of Markwell et al (1981). Ten micrograms of lysate protein was separated by SDS polyacrylamide gel electrophoresis (SDS-PAGE) using a 10% polyacrylamide gel and electroblotted onto a polyvinylidene difluoride membrane (Du Pont, Boston, MA, USA). After blockage of non-specific binding sites for 1 h with 5% non-fat milk in TPBS (PBS and 0.1% Tween-20), the membrane was incubated overnight at 4°C with anti-human rck/p54 antibody (Akao et al, 1995) at a dilution of 1:300. The membrane was then washed three times with TPBS, incubated further with alkaline phosphatase-conjugated goat anti-rabbit antibody (New England Biolabs, Beverly, MA, USA) at room temperature, and then washed three times with

© Cancer Research Campaign 1999

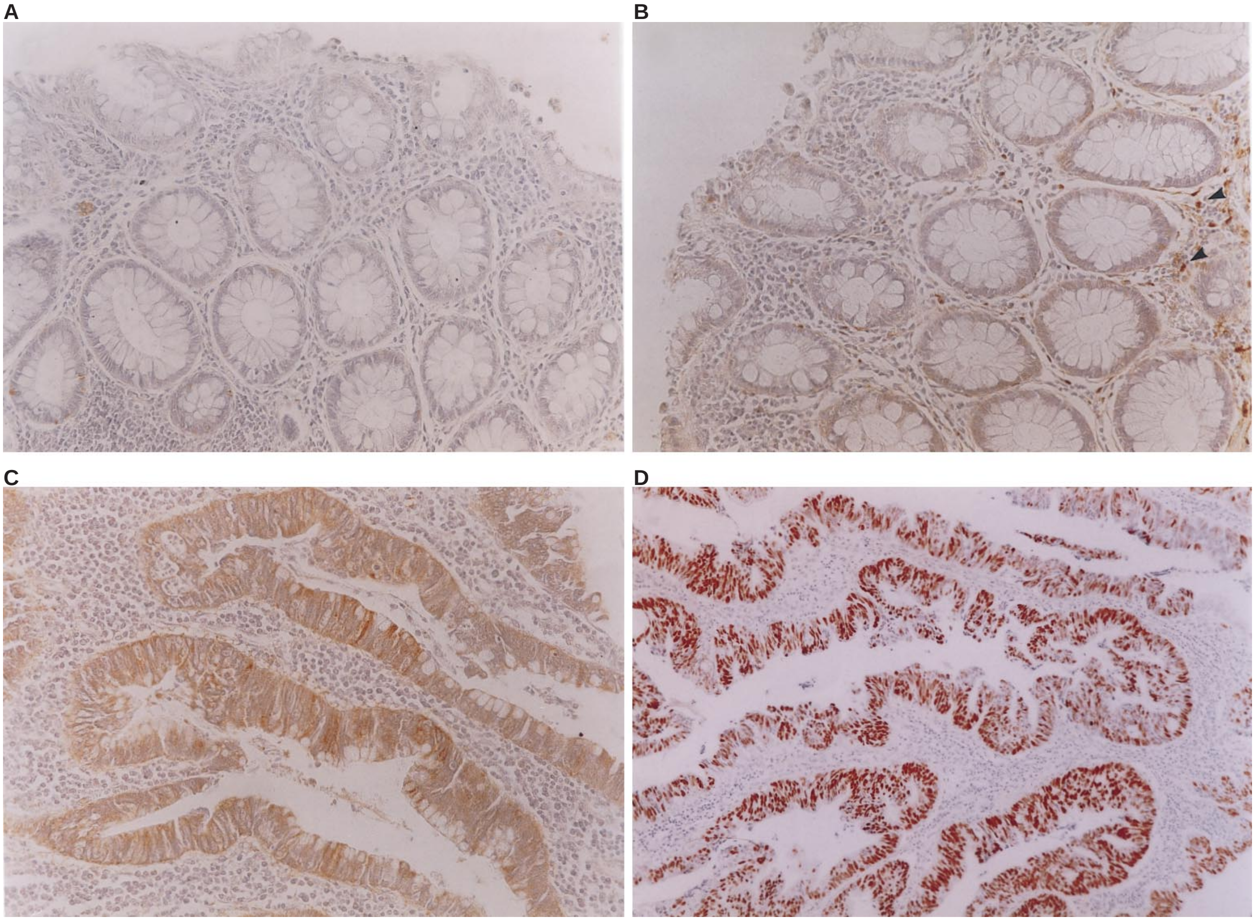

**Figure 1** Immunohistochemical staining of a human colorectal adenocarcinoma specimen (O-33) with anti-rck/p54 or anti-p53 antibody. (**A**) Normal colorectal mucosa from patient O-33 reacted with preimmune rabbit serum as a control. (**B**) Normal colorectal mucosa stained with anti-rck/p54 antibody. Note that faint rck/p54-specific staining is detectable on the basal side of glandular cells and in scattered cells such as histiocytes and fibroblasts in the mucosa. Arrowheads indicate the stromal rck/p54-staining cells. (**C**) Adenocarcinoma specimen stained with anti-rck/p54 antibody. It is evident that the adenocarcinoma cells that have deeply invaded are more intensely stained than normal cells in the mucosal region of (**B**). (**D**) Adenocarcinoma specimen stained with anti-p53 antibody. The nuclei of adenocarcinoma cells are diffusely stained

TPBS. The immunoblot was visualized by use of an enhanced chemiluminescence detection kit (New England Biolabs).

### RESULTS

The histopathological findings on 28 tissue samples of colorectal tumours that were examined immunohistochemically are summarized in Table 1. The median age of the 28 patients was 64 years (range 38–82 years old), and the subjects included 21 men and seven women. None had received radiation or chemotherapy prior to resection. rck/p54 overexpression was classified into two grades as 1+ or 2+. In 15 (54%) out of the 28 samples, rck/p54 was found to be overexpressed in adenocarcinoma and adenoma cells in comparison with its level in the normal tissue neighbouring the tumour in the same specimen (Table 1 and Figure 1 A–C). rck/p54 protein was found to be overexpressed in two cases (O-80 and O–81) of severe dysplastic adenomas. The correlation between rck/p54 expression and progression or types of cancers was not clear (Table 1). In normal tissues from the same samples, only a minute amount of rck/p54 was detected in the glandular cells of crypts; whereas free histiocytes, ganglion nerve cells and infiltrating lymphocytes were stained moderately. In both cases, rck/p54 displayed fine granular staining in the cytoplasm of the cells, which finding is in good agreement with the result of immunocytology of B-cells by laser microscopic analysis (Figure 1 B,C) (Akao et al, 1995). To examine the relationship between rck/p54 and mutant p53 protein (Finlay et al, 1988) in terms of expression, we also performed an immunohistochemical study on p53 protein in the O-33 specimen (Figure 1 C,D). Although the expression of mutant p53 protein did not correlate with that of rck/p54, taking all cases into account (Table 1), in five of the six rck/p54-positive (2+) cases, mutant p53 protein was detected.

To confirm the results of an immunohistochemical study on rck/p54, we performed Western blot analysis in four rck/p54-positive cases, viz. O-16, 33, 40 and 80. The quantative difference in rck/p54 expression was shown clearly between the homogenates prepared from normal and tumour tissues from each patient (Table 1 and Figure 2).

 *© Cancer Research Campaign 1999*

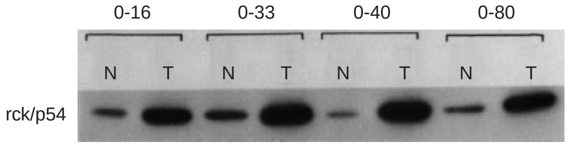

**Figure 2** Western blot analysis of rck/p54 in human colorectal normal mucosa and tumours. Ten micrograms of protein was electrophoresed on a 10% SDS-polyacrylamide gel. N indicates normal mucosal tissues of patients O-16, 33, 40 and 80 respectively; and T in matched samples from four patients indicates tumour tissues of patients O-16, 33, 40 and 80 respectively. O-80 is the only case of severe dysplastic adenoma. Note that the anti-rck/p54 antibody recognizes a 54-kDa molecule

### DISCUSSION

The present study demonstrated that rck/p54, a human DEAD box/RNA helicase protein, was overexpressed in approximately 54% of 28 cases of colorectal tumours studied by immunohistochemical analysis. Earlier, we reported that the *RCK* mRNA was shown to be expressed ubiquitously as judged from Northern blot analysis but that the amount of rck/p54 protein was translationaly regulated (Akao et al, 1995). In the brain, skeletal muscle and lung tissues, the expression of the protein was very low; but malignant cell lines that originated from these tissues, such as neuroblastoma, rhabdomyosarcoma and lung cancer cell lines, showed marked expression of it (Akao et al, 1995). Our findings on colorectal adenocarcinomas and adenomas are in good agreement with those on the expression pattern in those malignant cell lines. It is significant that the overexpression of rck/p54 was verified in the samples from patients with colorectal tumours. rck/p54 (2+) overexpression may be associated with mutant p53 protein (Finlay et al, 1988) expression, because five of the six rck/p54 (2+) cases expressed mutant p53 protein. It is of interest that rck/p54 was overexpressed in two cases of severe dysplastic adenomas. This finding suggests that rck/p54 may work at the early stage of malignant transformation. Based on these findings, rck/p54 of a DEAD box protein may possibly contribute to cell proliferation and carcinogenesis by facilitating the translation of some oncogene(s) or growth-associated gene(s) in colorectal tumours.

The DEAD box protein MrDb was reported to be one of the target proteins of the c-*myc* gene that positively functions in cell proliferation (Grandori et al, 1996). One possibility is that rck/p54 may be associated with the translation of mRNA from oncogenes such as c-*myc*, *E1A*, or other growth-related genes. We are now in the process of examining c-*myc* expression in the above samples of colorectal tumours.

It was reported that the overexpression of eIF-4E, which is a translation initiation factor forming a ternary complex with the eIF-4A DEAD box protein and p220, caused cell proliferation and malignant transformation (Lazaris-Karatzas et al, 1990). However, although the mechanism by which rck/p54 plays a role in transformation is not clear, it is postulated that high levels of rck/p54 may lead to the translation of mRNAs that are normally translationally repressed. Our finding suggests that the overexpression of rck/p54 could contribute to cell proliferation and malignant transformation at the translational level. It is still to be determined what gene(s) is facilitated in terms of translation of its mRNA as a step toward carcinogenesis.

### ACKNOWLEDGEMENT

This work was supported in part by a grant-in-aid for scientific research from the Ministry of Education, Science, Sports and Culture of Japan.

### REFERENCES

Akao Y, Seto M, Takahashi T, Kubonishi I, Miyoshi I, Nakazawa S, Tsujimoto Y, Croce CM and Ueda R (1991) Molecular cloning of the chromosomal breakpoint of a B-cell lymphoma with the t(11;14)(q23;q32) chromosomal translocation. *Cancer Res* **51**: 1574–1576

Akao Y, Seto M, Yamamoto K, Iida S, Nakazawa S, Inazawa J, Abe T, Takahashi T and Ueda R (1992) The *RCK* gene associated with t(11;14) translocation is distinct from the *MLL/ALL-1* gene with t(4;11) and t(11;19) translocations. *Cancer Res* **52**: 6083–6087

Akao Y, Marukawa O, Morikawa H, Nakao K, Kamei M, Hachiya T and Tsujimoto Y (1995) The rck/p54 candidate proto-oncogene product is a 54-kilodalton D-E-A-D box protein differentially expressed in human and mouse tissues. *Cancer Res* **55**: 3444–3449

Chuang R-Y, Weaver PL, Liu Z and Chang T-H (1997) Requirement of the DEAD-box protein Ded1p for messenger RNA translation. *Science* **275**: 1468–1471

Finlay CA, Hinds PW, Tan T-H, Eliyahu D, Oren M and Levine AJ (1988) Activating mutations for transformation by p53 produce a gene product that forms an hsp 70-p53 complex with an altered half-life. *Mol Cell Biol* **8**: 531–539

Grandori C, Mac J, Siebelt F, Ayer DE and Eisenman RN (1996) Myc-Max heterodimers activate a DEAD box gene and interact with multiple E box-related sites in vivo. *EMBO J* **15**: 4344–4357

Ladomery M, Wade W and Sommerville J (1997) Xp54, the *Xenopus* homologue of human RNA helicase p54, is an integral component of stored mRNP particles in oocytes. *Nucleic Acids Res* **25**: 965–973

Lazaris-Karatzas A, Montine KS and Sonnenberg N (1990) Malignant transformation by an eukaryotic initiation factor subunit that binds to mRNA 5′ cap. *Nature* **345**: 544–547

Markwell MAK, Hass SM, Tolbert NE and Bieber LL (1981) Protein determination in membrane and lipoprotein samples: manual and automated procedures. In *Methods in Enzymology*, Lowenstein, ML (ed), pp. 296–303 Academic Press: New York

Rabbitts TH (1994) Chromosomal translocations in human cancer. *Nature* **372**: 143–149

Rosen F, Edery I, Meerovitch K, Dever TE, Merrick WC and Sonenberg N (1990) Bidirectional RNA helicase activity of eukaryotic translation initiation factors 4A and 4F. *Mol Cell Biol* **3**: 1134–1144

*© Cancer Research Campaign 1999*
